# Hyperglycemia in bacterial meningitis: a prospective cohort study

**DOI:** 10.1186/1471-2334-9-57

**Published:** 2009-05-08

**Authors:** Ewout S Schut, Willeke F Westendorp, Jan de Gans, Nyika D Kruyt, Lodewijk Spanjaard, Johannes B Reitsma, Diederik van de Beek

**Affiliations:** 1 Department of Neurology, Center of Infection and Immunity Amsterdam (CINIMA), Academic Medical Center, Amsterdam, the Netherlands; 2 Department of Medical Microbiology, Center of Infection and Immunity Amsterdam (CINIMA), Academic Medical Center, Amsterdam, the Netherlands; 3 Netherlands Reference Laboratory for Bacterial Meningitis, Center of Infection and Immunity Amsterdam (CINIMA), Academic Medical Center, Amsterdam, the Netherlands; 4 Department of Clinical Epidemiology and Biostatistics, Center of Infection and Immunity Amsterdam (CINIMA), Academic Medical Center, Amsterdam, the Netherlands

## Abstract

**Background:**

Hyperglycemia has been associated with unfavorable outcome in several disorders, but few data are available in bacterial meningitis. We assessed the incidence and significance of hyperglycemia in adults with bacterial meningitis.

**Methods:**

We collected data prospectively between October 1998 and April 2002, on 696 episodes of community-acquired bacterial meningitis, confirmed by culture of CSF in patients >16 years. Patients were dichotomized according to blood glucose level on admission. A cutoff random non-fasting blood glucose level of 7.8 mmol/L (140 mg/dL) was used to define hyperglycemia, and a cutoff random non-fasting blood glucose level of 11.1 mmol/L (200 mg/dL) was used to define severe hyperglycemia. Unfavorable outcome was defined on the Glasgow outcome scale as a score <5. We also evaluated characteristics of patients with a preadmission diagnosis of diabetes mellitus.

**Results:**

69% of patients were hyperglycemic and 25% severely hyperglycemic on admission. Compared with non-hyperglycemic patients, hyperglycemia was related with advanced age (median, 55 yrs *vs. *44 yrs, P < 0.0001), preadmission diagnosis of diabetes (9% *vs. *3%, P = 0.005), and distant focus of infection (37% *vs. *28%, P = 0.02). They were more often admitted in coma (16% *vs. *8%; P = 0.004) and with pneumococcal meningitis (55% *vs. *42%, P = 0.007). These differences remained significant after exclusion of patients with known diabetes. Hyperglycemia was related with unfavorable outcome in a univariate analysis but this relation did not remain robust in a multivariate analysis. Factors predictive for neurologic compromise were related with higher blood glucose levels, whereas factors predictive for systemic compromise were related with lower blood glucose levels. Only a minority of severely hyperglycemic patients were known diabetics (19%). The vast majority of these known diabetic patients had meningitis due to *Streptococcus pneumoniae *(67%) or *Listeria monocytogenes *(13%) and they were at high risk for unfavorable outcome (52%).

**Conclusion:**

The majority of patients with bacterial meningitis have hyperglycemic blood glucose levels on admission. Hyperglycemia can be explained by a physical stress reaction, the central nervous system insult leading to disturbed blood-glucose regulation mechanisms, and preponderance of diabetics for pneumococcal meningitis. Patients with diabetes and bacterial meningitis are at high risk for unfavorable outcome.

## Background

Hyperglycemia has been reported to be associated with poor outcome in several infectious and neurological disorders, such as sepsis, stroke and severe head injury. [1-4] In bacterial meningitis, one study showed no association between hyperglycemia on admission and mortality, but this study was retrospective and involved low numbers of patients.[5] Acute hyperglycemia has been associated with an increase of circulating cytokine concentrations and with an impaired ability to oppose infection.[6] Intensive insulin therapy has shown to prevent morbidity and reduce mortality in critically ill patients.[7,8] In addition, it has been shown to have strong anti-inflammatory effects which could be of potential importance in patients with severe bacterial meningitis.[9]

In this nationwide prospective cohort study we assessed the prevalence and prognostic value of hyperglycemia on admission in patients presenting with community-acquired bacterial meningitis. We also evaluated characteristics of patients with a preadmission diagnosis of diabetes mellitus.

## Methods

The Dutch Meningitis Cohort Study is a prospective nationwide observational cohort study of 696 adults with community-acquired bacterial meningitis in the Netherlands, carried out between October 1998 and April 2002. Inclusion and exclusion criteria have been described extensively elsewhere.[10] In summary, all patients were aged over 16 years and had cerebrospinal fluid (CSF) culture proven bacterial meningitis. In total, 696 episodes of community-acquired bacterial meningitis occurred in 671 patients; 25 patients had a second episode of bacterial meningitis. Informed consent was obtained from participating patients or their legally authorized representatives. Information, including blood glucose values, was collected by means of a case record form. Neurological examination was performed on discharge and the patient's outcome was classified as unfavorable (defined by a Glasgow Outcome Scale score of 1 to 4 points at discharge) or favorable (a score of 5).[10,11] The Glasgow Outcome Scale is a well-validated measurement scale with scores varying from 1 (indicating death) to 5 (good recovery).[12] The study was performed in accordance with the Dutch privacy legislation and was approved by our ethics committee. Written informed consent to use data made anonymous was obtained from the patient (if possible) or from the patient's legal representative.

The criteria of the American Diabetes Association were used to define hyperglycemia in non-fasting blood samples.[13] In these guidelines non-fasting blood glucose levels of >7.8 mmol/L (>140 mg/dL) are defined as "hyperglycemia" and ≥ 11.1 mmol/L (>200 mg/dL) as diabetic values (in our paper referred to as "severe hyperglycemia"). We did not measure glycosylated hemoglobin (HbA1C) in our patients and had no information on formal glucose tolerance testing.

For the comparison of non-normally distributed variables nonparametric testing, Mann-Whitney *U*, χ^2^, or Fisher exact tests were used. We used logistic regression analysis to calculate OR and 95% CI to assess the strength of the association between potential risk factors and unfavorable outcome or hyperglycemia. Based on previous research and pathophysiological interest, 10 most relevant risk factors for outcome or blood glucose levels were selected.[14] Although the median percentage of missing values for individual variables in our study was low (2%), data were complete on all potential predictors in 408 out of 696 episodes (59%). Complete case analysis would potentially hamper the power of the multivariate models and, in addition, might lead to biased results. Therefore, we used the multiple imputation method to replace each missing value with a set of plausible values that represent the uncertainty about the right value to impute. By repeating this process several times, the uncertainty in the precision is properly taken into account.[15] The final estimates of the multivariate model were obtained by combining the results of 10 rounds of imputations. Analyses were also performed on the non-imputed dataset; in all presented analyses point estimates were similar as using the nonimputed dataset, with somewhat wider confidence intervals. Population description is done with medians and interquartile ranges (IQR). All analyses were performed using SAS software, version 9.11 (SAS Institute) and a 2-tailed *P *value below 0.05 was considered as significant.

## Results

Serum blood glucose levels were measured on admission in 682 of 696 episodes (98%). Hyperglycemia was present in 470 episodes (69%; Table 1), including severe hyperglycemia in 171 (25%). Two patients had a blood glucose level <4 mmol/L (<72 mg/dL); both patients had a preadmission diagnosis of diabetes. Patients with hyperglycemia were older than non-hyperglycemic patients and were more likely to have a preadmission diagnosis of diabetes. On the other hand, only a minority of the severely hyperglycemic patients were known diabetics (19%). Before admission, seizures were more often present among hyperglycemic patients as compared with non-hyperglycemic patients; however, this was not true for severe hyperglycemia.

**Table 1:** Features in adults with and without hyperglycemia or severe hyperglycemia

	Hyperglycemia glucose in mmol/L (mg/dL)			Severe hyperglycemia glucose in mmol/L (mg/dL)		
				
	<7.8 (140) (n = 212)	≥ 7.8 (140) (n = 470)	P	<11.1 (200) (n = 511)	≥ 11.1 (200) (n = 171)	P
Age	44 (24–64)	55 (37–68)	<0.001	48 (29–65)	59 (45–70)	<0.0001
Seizures	2/206 (1%)	29/447 (7%)	0.001	20/493 (4%)	11/160 (7%)	0.2
Distant infection focus *	60/212 (28%)	175/470 (37%)	0.02	155/511 (30%)	80/171 (47%)	<0.001
Diabetes	6/210 (3%)	40/466 (9%)	0.005	14/506 (3%)	32/170 (19%)	<0.001
Immunocompromised	26/211 (12%)	83/470(18%)	0.09	62/510 (12%)	47/171 (28%)	<0.001
GCS <14	127/211 (60%)	342/469 (73%)	0.001	327/510 (64%)	142/170 (84%)	<0.001
GCS <8 (coma)	17/211 (8%)	77/469(16%)	0.004	58/510 (11%)	36/170 (21%)	0.002
DBP < 60 mmHg	32/201 (16%)	29/457 (6%)	<0.001	58/490 (12%)	3/168 (2%)	<0.001
HR > 120 bpm	27/212 (13%)	48/470 (10%)	0.3	53/511 (10%)	22/171 (13%)	0.4
T >38.0	154/200 (77%)	357/465(77%)	1.0	380/496 (77%)	131/169 (94%)	0.8
Focal neurologic deficits^#^	67/212 (32%)	161/470 (34%)	0.5	162/511 (32%)	66/171 (39%)	0.1
*S. pneumoniae*	88/212 (42%)	256/470 (55%)	0.007	228/511 (45%)	116/171 (68%)	<0.001
CSF white cell count <1000	58/197 (29%)	76/436 (17%)	0.001	104/474 (22%)	30/159 (19%)	0.4
Platelet cell count	175 (121–237)	189 (147–240)	0.03	180 (139–236)	198 (150–253)	0.02
Positive blood culture	121/178(68%)	276/424 (65%)	0.5	295/447(66%)	102/155 (66%)	1.0
Unfavorable outcome	65/212 (31%)	163/470 (35%)	0.3	156/511 (31%)	72/171 (42%)	0.008
Mortality	38/212 (18%)	102/470 (22%)	0.3	96/513 (19%)	44/169 (26%)	0.05

Numbers are number/number assessed (percentage) or median (interquartile range) unless otherwise indicated. GCS denotes Glasgow Coma Scale, DPB diastolic blood pressure, HR hear rate, T bodily temperature (grade Celsius), CSF cerebrospinal fluid. * Defined as otitis/sinusitis or pneumonia. #Defined as cranial nerve palsies, aphasia, monoparesis or hemiparesis

On admission, patients with hyperglycemia were more likely to have a distant focus of infection (Table 1), mainly otitis or sinusitis. When patients with a preadmission diagnosis of diabetes were excluded, no association was found with otitis or sinusitis (P = 0.14), although there was such an association for severely hyperglycemic patients (P = 0.004). Patients with hyperglycemia were more likely to present with a change in mental status or in a coma. This difference remained significant after exclusion of diabetic patients (change in mental status: 72% *vs. *60%, P = 0.003). Lumbar puncture was performed in all episodes and CSF culture yielded *S. pneumoniae *in a higher proportion of episodes with hyperglycemia as compared with normal glucose levels. Interestingly, patients with hyperglycemia were less likely to present sign of systemic compromise: smaller proportions presented with a diastolic blood pressure <60 mm Hg, or CSF white blood cell counts <1000 cells/mm^3^, and they had higher platelet counts. CSF glucose levels were measured in 617 patients (89%) and correlated with blood glucose levels (Pearson's, P < 0.001). Median glucose CSF/serum ratio was 0.09 (IQR, 0.02–0.31) for patients with favorable outcome (GOS score 5, n = 459), and 0.05 (IQR, 0.01–0.21) for patients with unfavorable outcome (GOS score < 5, n = 237; P = 0.01). No statistically significant difference in glucose CSF/serum ratio was seen between patients who died (n = 143) and those who survived (n = 553, P = 0.24).

The overall number of patients with an unfavorable outcome was 228 (33%). Mortality was significantly higher in severely hyperglycemic patients (26% vs. 19%, P = 0.05). This association remained significant after exclusion of patients with a preadmission diagnosis of diabetes (40% vs. 30%, P = 0.03).

We performed a univariate analysis on the raw dataset, followed by a multivariate regression analysis to identify factors predictive for unfavorable outcome (Table 2). We also included serum glucose in this model. We identified several (known) factors for unfavorable outcome: *S. pneumoniae *as the causative microorganism, advanced age, a low score on the Glasgow Coma Scale, the presence of a focal neurologic deficit, a low platelet count and a low CSF leukocyte count. In the multivariate analysis serum glucose was no longer predictive of outcome.

**Table 2:** Univariate and Multivariate analysis of factors associated with unfavorable outcome

Characteristic	Odds ratio (95% CI) (univariate)	P	Odds ratio (95% CI) (multivariate)	P
Age, yrs	1.47 (1.35–1.62)	<0.0001	1.16 (1.03–1.16)	0.01
Known diabetes	2.24 (1.23–4.11)	<0.01	0.96 (0.42–2.16)	0.9
Immunocompromised, not diabetes	2.92 (1.76–4.90)	<0.0001	1.15 (0.61–2.17)	0.7
Heart rate				
< 60 bpm	2.26 (0.73–6.58)	<0.01	4.95 (1.09–22.45)	0.04
60–90 bpm	1.00 (reference)		1.00 (reference)	
>90–120 bpm	1.62 (1.09–2.44)		1.23 (0.75–2.01)	0.4
>120 bpm	3.96 (2.51–6.31)	<0.001	2.44 (1.35–4.43)	0.003
Diastolic blood pressure	0.97 (0.92–1.01)	0.01	0.95 (0.90–1.01)	0.09
Glasgow Coma Scale	0.82 (0.78–0.86)	<0.001	0.87 (0.81–0.93)	<0.001
Distant focus of infection*	2.05 (1.48–2.84)	<0.0001	1.04 (0.67–1.63)	0.9
Focal neurologic deficits^#^	2.50 (1.80–3.48)	<0.0001	1.93 (1.28–2.90)	0.002
Blood glucose	0.87 (0.69–1.07)	0.001	0.93 (0.75–1.16)	0.5
Log platelet count	0.62 (0.50–0.76)	<0.001	0.62 (0.48–0.81)	<0.001
Log CSF leukocyte count	0.80 (0.75–0.85)	<0.001	0.80 (0.74–0.87)	<0.001
Causative organism				
*S. pneumoniae*	7.65 (5.02–12.00)	<0.0001	4.95 (2.69–9.12)	<0.001
*N. meningitidis*	1.00 (reference)		1.00 (reference)	
Other	3.98 (2.22–7.17)	<0.001	2.38 (1.12–5.04)	0.02

* Defined as otitis/sinusitis or pneumonia. # Defined as cranial nerve palsies, aphasia, monoparesis or hemiparesis. Bpm denotes beats per minute, CSF cerebrospinal fluid.

As a next step we undertook a multivariate analysis of factors related to blood glucose levels. The following factors were associated with a rise of serum glucose: a preadmission diagnosis of diabetes (an extra 5.30 mmol/L or 95.36 mg/dL of glucose if present), age (every year added an extra 0.02 mmol/L or 0.31 mg/dL of glucose, P = 0.03), the presence of a distant focus of infection (an extra 0.88 mmol/L or 15.77 mg/dL of glucose if present, P = 0.004), the presence of immunocompromised states other than diabetes (an extra 0.99 mmol/L or 17.84 mg/dL of glucose if present, P = 0.03), diastolic blood pressure (every 1 mm Hg added an extra 0.15 mmol/L or 2.62 mg/dL of glucose, P < 0.001), sum score on the Glasgow Coma Scale (every decrease by one point added an extra 0.15 mmol/L or 2.77 mg/dL of glucose, P < 0.001) and CSF leukocyte count (every 10-fold decrease added an extra 0.16 mmol/L or 2.86 mg/dL of glucose, P < 0.001). Platelet counts or infection with *S. pneumoniae *were not related to blood glucose levels in this analysis

A preadmission diagnosis of diabetes was present in 46 patients (Table 3); 87% of these patients were hyperglycemic on admission and 70% were severely hyperglycemic. The median age was 69 years and predisposing conditions other than diabetes were present in a large proportion of patients (39%). Patients had severe illness as reflected in low levels of consciousness and high rates of focal neurologic deficits. Overall, the clinical presentation was not "atypical" (i.e., lacking the "classical" clinical symptoms of fever, headache and a decreased level of consciousness) and the vast majority of patients had pneumococcal meningitis (67%). Other causative organisms were *Listeria monocytogenes *(13%), group B streptococci (4%), and *Escherichia coli *(2%). The rate of unfavorable outcome among diabetic patients was high (52%).

**Table 3:** Characteristics of diabetic patients with meningitis (n = 46)

Characteristics	value	Characteristics	value
Median age in years (IQR)	69 (59–74)	Laboratory features	
Symptoms <24 hours	30/45 (65%)	CSF WBC <1000/mm^3^	10/46 (23%)
Predisposing conditions		Blood glucose ≥ 7.8 mmol/L (≥ 140 mg/dL)	40 (87%)
Distant focus of infection*	33/46 (72%)	Blood glucose ≥ 11.1 mmol/L (≥ 200 mg/dL)	32 (70%)
Otitis/sinusitis	15/46 (33%)	Positive blood culture	25/46 (57%)
Pneumonia	5/46 (11%)	Causative organism	
Symptoms and signs		*S. pneumoniae*	31/46 (67%)
Headache	33/41 (81%)	*N. meningitidis*	6/46 (13%)
Neck stiffness	34/44 (77%)	*Listeria monocytogenes*	6/46 (13%)
Temperature >38°C	38/46 (83%)	Group B streptococci	2/46 (4%)
GCS <14	35/45 (78%)	*Escherichia coli*	1/46 (2%)
Score on GCS <8	14/44 (31%)	Favorable outcome	22/46 (48%)
Focal neurologic deficits^#^	24/46 (52%)	Death	15/46 (33%)

* Defined as otitis/sinusitis or pneumonia. # Defined as cranial nerve palsies, aphasia, monoparesis or hemiparesis. IQR denotes interquartile rate, GCS Glasgow Coma Scale, WBC white blood cell count

## Discussion

Our study shows that hyperglycemia is a very common finding in patients presenting with bacterial meningitis. One out of four patients in our cohort had severe hyperglycemia which was related with unfavorable outcome in a univariate analysis (P = 0.008). However, the association between glucose levels and unfavorable outcome did not remain robust in a multivariate analysis.

Many of the factors predictive for unfavorable outcome were also predictive for severe hyperglycemia on admission. High blood glucose levels seem to be an epiphenomenon of disease severity and physical stress, at least partly. Factors predictive for systemic compromise are strong predictors of unfavorable outcome in adults with bacterial meningitis.[8] This relation is comparable with any other critical illness. However, some of the well known factors predictive for unfavorable outcome in bacterial meningitis, such as low CSF white cell counts, low diastolic blood pressure, and low thrombocyte counts, were not related to high glucose serum levels. On the contrary, these factors had an inverse relation with serum blood glucose levels. This finding could not be explained by differences in causative bacterial agents between groups. Our results therefore emphasize the importance of the insult to the central nervous system, rather than the systemic insult in patients with bacterial meningitis, leading to disturbed blood-glucose regulation mechanisms. This effect has been described previously in patients after stroke and might be caused by effects on central autonomic control sites.[16,17]

Diabetes was by far the strongest predictor for higher glucose levels on admission. The overall proportion of patients with a pre-admission diagnosis of diabetes was 7%. However, this percentage might have been underestimated since many patients had diabetic blood glucose levels on admission. The incidence of diabetes among adults with bacterial meningitis has been reported to be as high as 39% in Taiwan.[18] The meningitis population in Taiwan differed markedly from our cohort with respect to the causative organisms. In Taiwan, *Klebsiella pneumoniae *was the most common causative organism in both diabetic and non-diabetic patients, whereas no infection with *S. pneumoniae *occurred in any of the diabetic patients. By contrast, in our cohort *S. pneumoniae *was the most common causative microorganism and was significantly more common in patients with hyperglycemia (55%) than in patients without hyperglycemia (42%). Differences in microbial epidemiology, innate immunity and health care might account for these differences.

As in stroke, hyperglycemia could be a measure of previously unrecognized insulin resistance or diabetes.[19] This idea is supported by the observation that predictors for hyperglycemia were also associated with diabetes before admission: older age, other immunocompromise than diabetes (use of immunosuppressive medication, like corticosteroids), and a distant focus of infection.

The majority of patients with a pre-admission diagnosis of diabetes had pneumococcal meningitis (67%). Diabetes is a well known risk factor for invasive pneumococcal diseases. In the U.S. and some European countries, physicians are advised to vaccinate diabetic patients with the 23-valent pneumococcal polysaccharide vaccine [20-22]. The 23-valent pneumococcal polysaccharide vaccine would have provided coverage for 87% of all causative pneumococcal strains in the Dutch Meningitis Cohort Study.[23] Vaccine efficacy however, is likely less than 60%[24] and no randomized controlled clinical trials using this vaccine have yet been carried out in diabetic patients to support this recommendation.

This study has several limitations. First, only patients who had a positive CSF culture were included. Negative CSF cultures are estimated to occur in 11 to 30% of patients with bacterial meningitis.[10,25] No significant differences in clinical presentation have been reported between culture-positive bacterial meningitis and those with culture-negative bacterial meningitis.[25,26] It is therefore unlikely that this factor confounded our results. Second but more importantly, we used a single random blood glucose measurement on admission to define hyperglycemia. As can be seen in stroke[27] and subarachnoid hemorrhage[3], persistent hyperglycemia or persistently high levels of fasting blood glucose may be more important than hyperglycemia on admission and might have an independent effect on outcome, which we were not able to assess because follow-up blood glucose concentrations were unavailable. We also did not measure glycosylated hemoglobin (HbA1C) in our patients. This is an important limitation of our study. Finally, we have no data on the adequacy of antihyperglycemic treatment and treatment-related hypoglycemia.

Although the exact nature of the relation between severe hyperglycemia and outcome in bacterial meningitis remains uncertain, data of previous studies including critically ill patients suggest that severe hyperglycemia should be treated, also in adults presenting with bacterial meningitis. Based on these previous studies, we recommend aiming for blood glucose levels below 10.0 mmol/L (180 mg/dL) throughout the clinical course, while trying to avoid hypoglycemia. The increased risk of hypoglycemia has been one of the major concerns put forward with regard to intensive insulin therapy.[28] A recently published study even found an increased mortality and a higher incidence of hypoglycemia in critically ill patients who where treated with intensive glucose control as compared to those who were treated with conventional glucose control.[29] Although we were unable to assess the frequency and severity of treatment-related hypoglycemia in our cohort, hypoglycemia can be life threatening and hypoglycemic symptoms are not easily recognized in patients with acute bacterial meningitis. Severe hypoglycemia or prolonged hypoglycemia can lead to convulsions, coma, and irreversible brain damage. In the single-center prospective randomized controlled trials from Leuven, the risk of hypoglycemia (glucose of ≤ 2.2 mmol/L or ≤ 40 mg/dL) increased from 0.8% to 5.1% in the surgical ICU trial and from 3.1% to 18.7% in the medical ICU trial.[7,8] In those ICU based trials, insulin adjustments were based on measurements of blood glucose levels in arterial blood, at one- to four-hour intervals, and doses were administered by a team of intensive care nurses, assisted by a study physician. The question arises whether intensive insulin therapy is feasible, safe and effective when implemented outside of the ICU, where most patients with acute bacterial meningitis are managed. Previous studies have shown that control of hyperglycemia in patients admitted to a stroke unit remains difficult.[19] However, in light of the growing evidence of the detrimental effects of sustained hyperglycemia in other neurological diseases, physicians treating patients with bacterial meningitis should play a more active role in the control of blood glucose levels.

## Conclusion

The majority of patients with bacterial meningitis have hyperglycemic blood glucose levels on admission. Hyperglycemia can be explained by a physical stress reaction, the central nervous system insult leading to disturbed blood-glucose regulation mechanisms, and preponderance of diabetics for pneumococcal meningitis. Patients with diabetes and bacterial meningitis are at high risk for unfavorable outcome.

## Competing interests

The authors declare that they have no competing interests.

## Authors' contributions

ES participated in the analysis and interpretation of the data, and drafted the manuscript. WFW participated in the analysis and interpretation of the data, and writing of the manuscript. JdG participated in the data collection. NDK participated in writing the manuscript. LS participated in writing the manuscript. JBR participated in the analysis and interpretation of the data, and writing the manuscript. DvdB concepted and designed the study, collected the data, participated in the analysis and interpretation of the data, and drafted the manuscript. All authors read and approved the final manuscript.

## Author Information

Correspondence: Diederik van de Beek, MD, PhD, Department of Neurology and Center of Infection and Immunity Amsterdam (CINIMA), Academic Medical Center, University of Amsterdam, P.O. Box 22660, 1100DD Amsterdam, The Netherlands (d.vandebeek@amc.uva.nl).

## Pre-publication history

The pre-publication history for this paper can be accessed here:

http://www.biomedcentral.com/1471-2334/9/57/prepub
